# The Effect of Maleated Polypropylene on the Non-Isothermal Crystallization Kinetics of Wood Fiber-Reinforced Polypropylene Composites

**DOI:** 10.3390/polym10040382

**Published:** 2018-04-01

**Authors:** Chung-Wei Huang, Teng-Chun Yang, Ke-Chang Hung, Jin-Wei Xu, Jyh-Horng Wu

**Affiliations:** Department of Forestry, National Chung Hsing University, Taichung 402, Taiwan; tonyst61112@gmail.com (C.-W.H.); tcyang.04@nchu.edu.tw (T.-C.Y.); d9833004@mail.nchu.edu.tw (K.-C.H.); ecsgunro@gmail.com (J.-W.X.)

**Keywords:** wood fiber, maleated polypropylene (MAPP), polypropylene (PP), cooling rate, non-isothermal crystallization, crystallization kinetics

## Abstract

The influence of maleated polypropylene (MAPP) on the non-isothermal crystallization behavior of wood fiber (WF)-reinforced PP composites (WPCs) was investigated by a differential scanning calorimeter (DSC). The results showed that MAPP as a nucleation agent accelerated the crystallization rate of the PP matrix in WPC under the cooling process. The corresponding crystallization kinetics and activation energy were further analyzed using the Avrami method, Avrami–Ozawa method, Kissinger method, and Friedman method. The results demonstrated that MAPP significantly changed the crystal growth mechanism of the PP matrix to heterogeneous nucleation for acicular and tabular crystal growth during the annealing step. A remarkably lower cooling rate can achieve a certain relative crystallinity degree at the unit crystallization time for WPC with 3 wt % MAPP (WPC_M3_). Similarly, the lowest crystallization activation energy was observed for the WPC_M3_ among all WPCs by the Kissinger method. Furthermore, based on the Friedman method, the addition of MAPP easily caused the PP matrix to crystallize in the WPC at the initial stage of relative crystallinity.

## 1. Introduction

Over the last decade, wood fiber (WF)-reinforced polymer matrices, consisting of petroleum-based and renewable resources, have gained interest for the manufacture of products in a number of fields, such as fencing, decking, and automotive components, due to sustainability, relatively high strength and stiffness, low maintenance and cost, and good durability [1,2,3,4,5]. However, the incompatibility between hydrophilic lignocellulosics and hydrophobic thermoplastics has been found to limit the applicability of wood–plastic composites (WPCs). This deficiency produces insufficient interfacial adhesion, which may lead to moisture intrusion and biological attack [6,7]; in addition, this incompatibility results in decreased mechanical properties due to poor stress transition from the matrix to the fibers [8,9]. To enhance the interfacial adhesion and to improve the properties of the composites, many physical and chemical approaches, such as the addition of coupling agents [8,9,10,11,12], silane treatment [10,13,14], alkaline treatment [13,15,16], esterification [15,17], and heat treatment [18,19], have been used. Under the consideration of usability and eco-effectiveness, the use of coupling agents has been attractive since some coupling agents can create significant bonds between the WF and the polymer matrix and improve the composite compatibility. Among the coupling agents, copolymers containing maleic anhydride, such as maleated polypropylene (MAPP), are commonly and widely used in WPCs [8,9,10,11,12]. Until recently, a hypothetical coupling mechanism of the wood–polymer interface was that anhydride molecules can react to form covalent bonds with the hydroxyl groups of the WF, and the other end of the maleic anhydrides of the copolymers entangle and cohere with the polymer matrix. This reaction makes the surface energy of the polypropylene (PP) matrix much closer to that of the WF, further resulting in better interfacial interactions between the PP matrix, WFs, and wettability [11,20,21]. Therefore, after the addition of MAPP as a coupling agent in WPCs, the improvement of their dimensional stability, mechanical properties, and impact strength has been reported in several studies [22,23,24,25]. To date, investigations on WPCs have primarily focused on the effects of various attributes to the performances of the composites, such as fiber type, fiber loading, and fiber modification. However, the quality and properties of the end products have been significantly influenced by the resulting microstructure, especially the crystallization of the polymer matrix, which is dependent on the thermal and press history during processing [26]. In addition, polymer composites are invariably solidified under non-isothermal conditions in industrial processing, e.g., extrusion and compression moldings. Therefore, information on the non-isothermal crystallization behavior of WPCs is still indispensable to understand the crystallization mechanism of the final composite material. Thus far, the kinetic aspects of the crystallization process have been examined by alternative methods, such as the Avrami theory, to investigate the non-isothermal crystallization behavior of polymer composites with various natural fibers [21,27,28,29,30]. On the other hand, a few studies have reported the crystallization kinetics of WPCs with a constant amount of coupling agent under non-isothermal conditions [29,31]. However, to the best of our knowledge, no clear indication of the effect of different MAPP concentrations on the non-isothermal crystallization behavior of WF/polymer composites has been obtained. Therefore, the aim of the present study was to examine the crystallization behaviors of WPCs with various amounts of MAPP under non-isothermal conditions by differential scanning calorimetry (DSC) analysis. Investigations of the nucleation mechanism and the required crystallization rate were analyzed by the Avrami method and the Avrami–Ozawa method, respectively. Additionally, the Kissinger method and Friedman method were applied to obtain the activation energy of non-isothermal crystallization.

## 2. Materials and Methods

### 2.1. Materials

PP pellets (Globalene 7633) were purchased from LCY Chemical Co. (Taipei, Taiwan), and their density, melting point (MP), and melt flow index (MFI) were 896 kg/m^3^, 170 °C, and 2 g/10 min, respectively. The commercially available coupling agent MAPP (density: 934 kg/m^3^; MP: 156 °C; MFI: 115 g/10 min) was purchased from Sigma-Aldrich Chemical Co. (St. Louis, MO, USA). WFs were prepared from a 52-year-old China fir (*Cunninghamia lanceolata*), which was kindly provided by the experimental forest of National Chung Hsing University (Nantou, Taiwan), via hammer-milling and sieving to obtain fibers between 16 and 24 mesh (1.00–0.71 mm). As shown in Table 1, the weight ratios of oven-dried WF, PP, and MAPP were 0/100/0, 60/40/0, 60/39/1, 60/37/3, 60/35/5, and 60/33/7 (wt %), designated Neat PP, WPC_0_, WPC_M1_, WPC_M3_, WPC_M5_, and WPC_M7_, respectively. Each composition was compounded at 200 °C for 5 min by a YKI-3 Banbury mixer (Goldspring Enterprise Inc., Taichung, Taiwan) at a rotor speed of 50 rpm and consequently extruded and pelleted into 4–5 mm diameters and 5–7 mm length pellets.

### 2.2. Differential Scanning Calorimetry Measurement

Neat PP and various WPC pellets were ground in an attrition mill, and a total of 3–5 mg of each sample was prepared directly in DSC aluminum pans with lid. The non-isothermal crystallization was executed in nitrogen at a flow rate of 20 mL/min using a PerkinElmer DSC-6 (Beaconsfield, UK). To eliminate the thermal and mechanical history and to ensure a nuclei-free melt, the first step was preliminary heating of the samples to 200 °C with a heating rate of 10 °C/min, and the temperature was held for 5 min. Afterward, the samples were cooled to 50 °C at different constant cooling rates of 2.5, 5, 10, 15, 20 and 25 °C/min. The exothermic flow curves as a function of temperature were recorded to analyze the non-isothermal crystallization kinetics. Two individual samples for each composition were tested. The PP crystallinity (*X*_c_) was obtained as a function of the cooling rate using the following expression:

(1) $$X_{c}\left(\%\right)=\frac{\Delta H_{m}}{\Delta H_{m}^{0}\times W_{p}}$$

where Δ*H*_m_ is the melting enthalpy for the sample at a given cooling rate, Δ*H*_m_^0^ denotes the melting enthalpy of 100% crystalline PP, the value of which is 209 J/g [32], and *W*_p_ denotes the weight percentage of the PP in the samples.

### 2.3. Crystallization Kinetics

The relative crystallinity (*X*) as a function of crystallization temperature (*T*) can be formulated based on the non-isothermal DSC data by using the following equation:

(2) $$X\left(\%\right)=\overset{T}{\underset{T_{0}}{\int }}\left(\frac{dH_{c}}{dT}\right)dT/\overset{T_{\infty }}{\underset{T_{0}}{\int }}\left(\frac{dH_{c}}{dT}\right)dT$$

where *T*_0_ and *T*_∞_ are the onset and offset temperatures of crystallization, respectively, and *dH*_c_/*dT* is the heat flow rate. The crystallization time (*t*) at a given cooling rate can be calculated as follows:

(3) $$t=\left(T_{0}-T\right)/\phi$$

where *T*_0_ is the onset temperature at crystallization time *t* = 0, *T* is the temperature at time *t*, and *ϕ* is the cooling rate.

The crystallization kinetics could be determined based on the Avrami (Equation (4)) [33,34,35] and Ozawa (Equation (5)) approaches [36] using the following equations:

(4) $$X\left(t\right)=1-e^{-Kt^{n}}$$

(5) $$-\ln\left(1-X\left(T\right)\right)=\frac{k\left(T\right)}{\phi^{m}}$$

where *X*(*t*) is the relative crystallinity at time *t*, *K* is the Avrami crystallization rate constant indicating nucleation and growth rate parameters, *n* denotes a mechanism constant that explains the nucleation and growing geometry of the crystallites, *X*(*T*) is the relative crystallinity at temperature *T*, *k*(*T*) is the cooling crystallization function, *m* is the Ozawa exponent, which depends on the nucleation mechanism and the crystal growth, and *ϕ* is the constant cooling rate. Furthermore, by taking a logarithm of both sides, Equations (4) and (5) can be converted into Equations (6) and (7), respectively:

(6) log[−ln(1−X(t))]=nlog t+log K

(7) log[−ln(1−X(T))]=log k(T)−mlog ϕ.

However, the Ozawa equation has been proven to be inappropriate for describing the non-isothermal crystallization kinetics of some polymer systems [37,38,39]. Therefore, by combining the Avrami and Ozawa equations, an equation related to the cooling crystallization rate *ϕ* and time *t* could be generated at a given degree of crystallization under dynamic crystallization conditions [40,41] as follows:

(8) ln ϕ=ln F(T)−αln t

where *F*(*T*) = [*k*(*T*)/*K*]^1/*m*^ denotes the value of the cooling rate at the unit crystallization time under a given degree of crystallinity, and *α* denotes the ratio of the Avrami exponent *n* to the Ozawa exponent *m*.

Moreover, the crystallization activation energy was calculated by the Kissinger equation (Equation (9)) and the Friedman equation (Equation (10)), and these equations are expressed as follows [42,43]:

(9) $$\frac{d\left[\ln\;\left(\phi /T_{p}^{2}\right)\right]}{d\left(1/T_{p}\right)}=\frac{-\Delta E}{R}$$

(10) $$\ln{\left(\frac{dX}{dt}\right)}_{X}=\ln\left[A_{X}f\left(X\right)\right]-\frac{E_{X}}{RT_{X}}$$

where *T*_p_ is the crystallization peak, *ΔE* is the activation energy, which characterizes the transport process of macromolecular segments to the surface of crystal growth, *R* is the universal gas constant, *X* is the relative degree of crystallinity, *t* is the time, *f*(*X*) is the function of the reaction mechanism, and *A_X_*, *E_X_*, and *T_X_* are the pre-exponential factor, the activation energy, and the temperature at a given *X* value, respectively.

## 3. Results and Discussion

### 3.1. Non-Isothermal Crystallization Behavior

**Figure 1:**
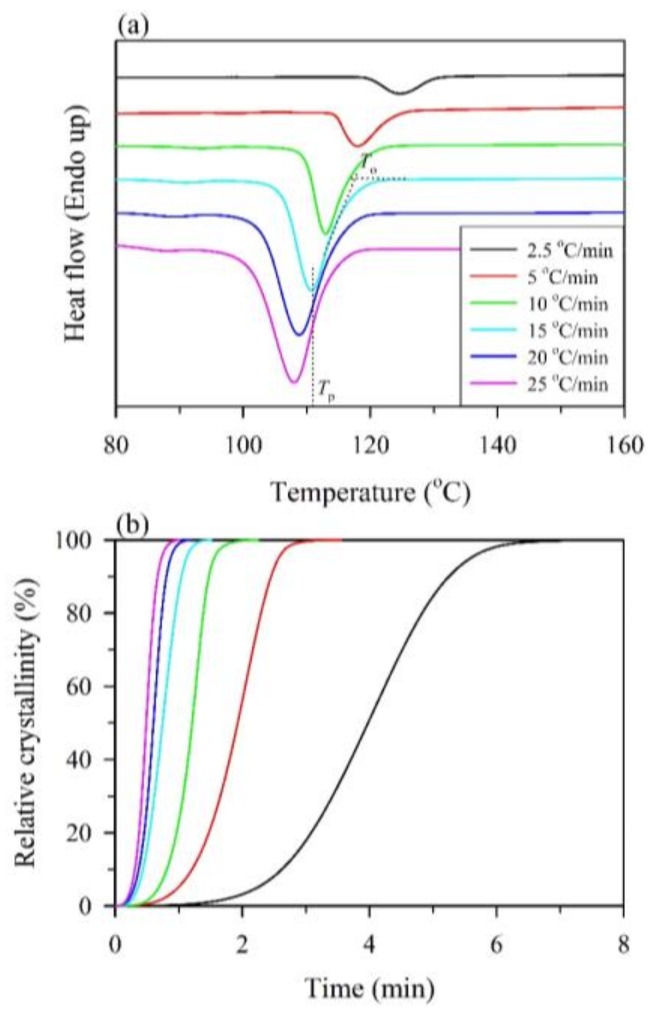
Non-isothermal crystallization behaviors of the neat PP. (**a**) Differential scanning calorimetry (DSC) thermograms. (**b**) Relative crystallinity as a function of time.

**Figure 2:**
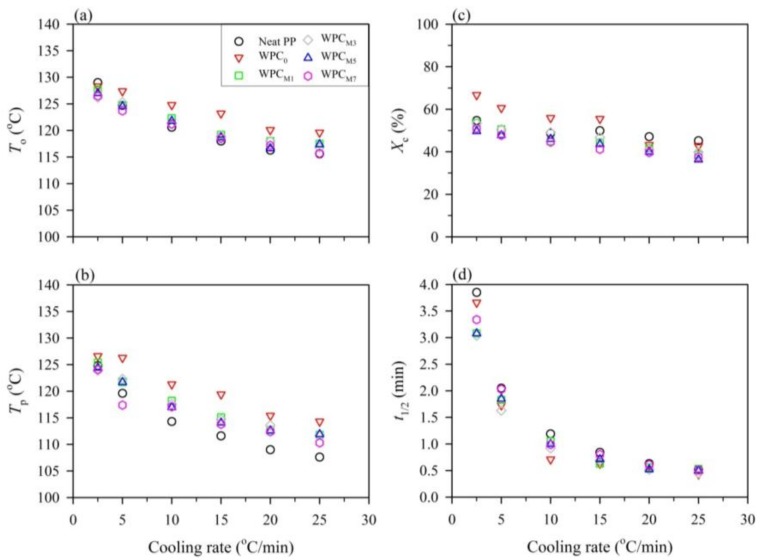
Non-isothermal crystallization parameters as a function of the cooling rate for the neat PP and wood–plastic composites (WPCs) with various maleated polypropylene (MAPP) concentrations. (**a**) The onset temperature (*T*_o_); (**b**) The crystallization peak (*T*_p_); (**c**) Crystallinity (*X*_c_); (**d**) The half-time (*t*_1/2_).

As shown in Figure 1a, the onset temperature (*T*_o_) and the crystallization peak (*T*_p_) can be obtained from the DSC exothermic curves of the non-isothermal crystallization of neat PP for a range of cooling rates from 2.5 to 25 °C/min. It can be seen that the *T*_o_ and *T*_p_ shifted to lower temperatures, and the range of crystallization temperatures broadened for all samples with an increasing cooling rate. This result is attributed to the fact that the motion of the PP chains lagged at higher cooling rates, and crystallization occurred at lower temperatures. In contrast, sufficient time to induce nuclei was available at lower cooling rates, resulting in the initiation of crystallization at higher temperatures [29,44]. In Figure 2a,b, at the given cooling rate, the *T*_o_ and *T*_p_ values of WPC_0_ and MAPP-containing WPCs were higher than those of neat PP, especially for the WPC_0_. These results indicated that the WF or MAPP could act as a heterogeneous nucleation agent for the crystallization of PP and accelerate the crystallization process. On the other hand, as shown in Figure 2c, the crystallinity (*X*_c_) value of the neat PP decreased from 54.7% (2.5 °C/min) to 45.2% (25 °C/min) with an increase in the cooling rate, and the other samples showed the same tendency. This result indicated that a higher crystallinity of the PP matrix was produced at a lower cooling rate. When WFs were incorporated in the PP matrix, the *X*_c_ value increased by 11–22%, especially at a lower cooling rate (<15 °C/min), indicating that the WF could significantly increase the crystallinity of the PP matrix. However, the *X*_c_ values of the MAPP-containing WPCs were slightly lower than those of the neat PP at various cooling rates. This result probably suggested that the transport of PP molecular chains was impeded at the crystal surface due to the high density of nuclei for PP with WF and MAPP [29,45].

Figure 1b shows the relative crystallinity (*X*) as a function of the crystallization time (*t*) for the neat PP at various cooling rates. It can be seen that the curves showed sigmoidal growth, and this trend is essentially the same for all samples with different cooling rates. This result clearly indicated the lag effect of the cooling rate on the crystallization [31]. In many previous studies [37,40,46], the trend of these curves was attributed to two crystallization processes including the initial stage of a fast crystallization process and the later stage of a slower crystallization process. During the later stage of the curve (Figure 1b), it can be seen that the crystallization rate significantly decreased, which resulted from the impingement of spherulites [37,40]. Additionally, at the slower cooling rate, the curve ramp was decreased compared to the higher cooling rate, indicating that the time for crystallization completion decreased with increasing cooling rates. This phenomenon was further determined for each sample by the half-time of crystallization (*t*_1/2_), which is defined as the period corresponding to an *X* of 50%. As shown in Figure 2d, the *t*_1/2_ values of all samples were reduced with increasing cooling rates. For instance, it can be seen that the *t*_1/2_ value of the neat PP decreased from 3.85 min at 2.5 °C/min to 0.52 min at 25 °C/min. Furthermore, the *t*_1/2_ value of various WPCs was lower than that of the neat PP, and the effect of MAPP is clearly seen in the case of lower cooling rates. At a cooling rate of 2.5 °C/min, the *t*_1/2_ value decreased to 3.66 min when WFs were added to the PP matrix (WPC_0_), and this value further decreased to 3.04 min as MAPP content increased up to 3 wt %. Once the MAPP content exceeded 3 wt %, however, an increased *t*_1/2_ value was observed, e.g., 3.34 min for WPC_M7_. This result is attributed to the fact that the rearrangement of molecular chains is hindered due to the greatest number of nucleation sites, resulting in a reduced crystallization rate of the PP matrix.

### 3.2. Kinetic Analysis with the Avrami Approach

**Figure 3:**
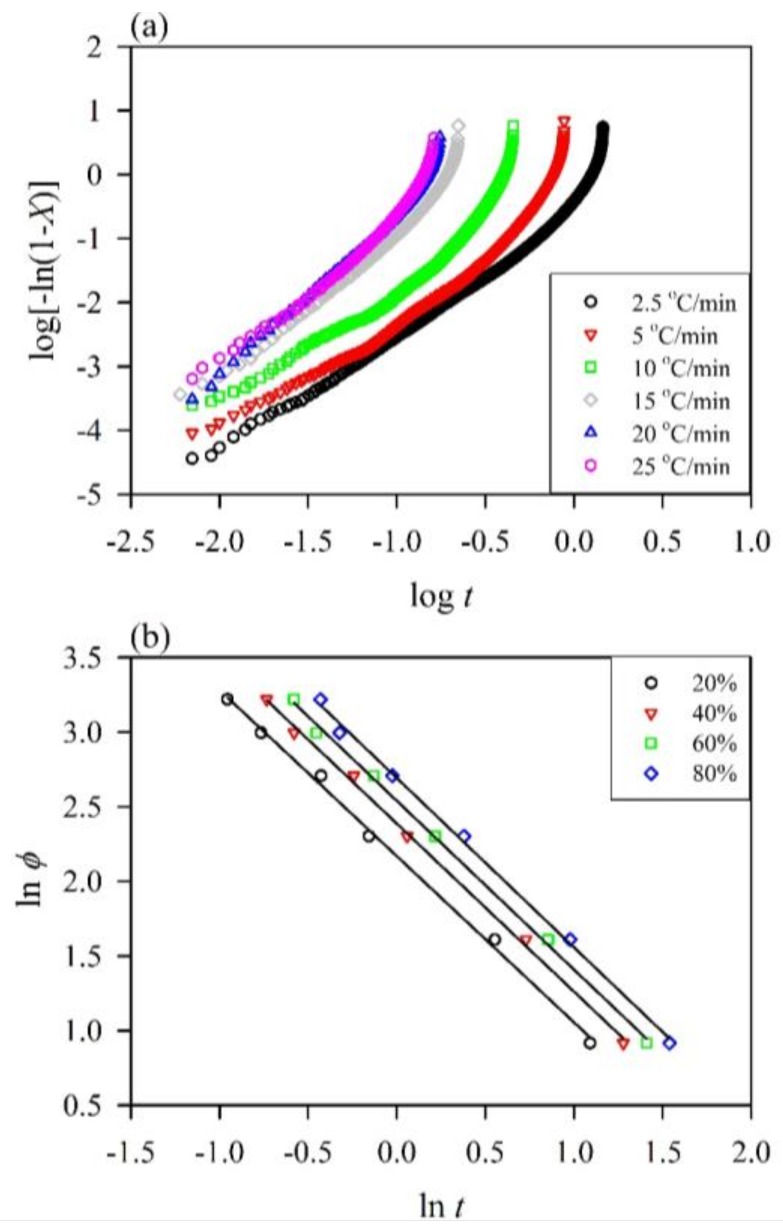
The non-isothermal crystallization kinetics of the neat PP. (**a**) Avrami plots of log[–ln(1–*X*)] versus log *t*; (**b**) Avrami–Ozawa plots of ln *ϕ* versus ln *t*.

The nucleation mechanisms were further analyzed with the Avrami approach. According to the assumption that the crystallization temperature is constant, the Avrami model is widely applied to the isothermal crystallization kinetics of polymers to describe the initial stage of crystallization. At a given cooling rate, a plot of log[–ln(1–*X*(*t*))] versus log *t* can be used to obtain the values of *n* and *K* from the slope of the linear portion and intercept of the curve according to Equation (6). As shown in Figure 3a, linear fits of the plots describing the initial stage of crystallization were conducted for the log[–ln(1–*X*(*t*))] values. A linear regression was performed on the slope for all samples as validated by the square of the correlation coefficient (*R*^2^) value which was greater than 0.94 (Table 2). The fitting lines of each section were nearly parallel for the neat PP, implying that the nucleation mechanism and geometry of crystal growth at different cooling rates were similar, and the same tendency for various WPCs was observed (not shown). On the other hand, the *n* values ranged from 1.67 to 1.99 for the neat PP at different cooling rates, suggesting that its non-isothermal crystallization corresponded to acicular crystal growth with homogeneous nucleation [47]. The following *n* values of all WPCs were in the range of 1.28–2.04, suggesting that their non-isothermal crystallization mechanisms were acicular and tabular crystal growth with heterogeneous nucleation [30,47]. These results indicated that the addition of WF and MAPP significantly changed the nucleation mechanism of the PP matrix in WPCs under the crystallization process. Moreover, the crystallization rate constant (*K*) was corrected to obtain the crystallization rate (*K*_J_) as a function of the cooling rate (*ϕ*) according to the following equation [48]:

(11) $$\log\;K_{J}=\log\;K/\phi .$$

As shown in Table 2, the *K*_J_ values for all samples markedly increased with increasing cooling rates up to 10–15 °C/min, indicating that the highest cold crystallization rate occurs at 10–15 °C/min. Furthermore, it can be seen that the WPC_M3_ almost exhibited the highest *K*_J_ value among all WPCs at any given cooling rate. The *K*_J_ values were in the ranges of 0.67–1.07, 0.52–1.07, 0.83–1.08, 0.55–1.04, and 0.42–1.12 for WPC_0_, WPC_M1_, WPC_M3_, WPC_M5_, and WPC_M7_, respectively, at cooling rates of 2.5–25 °C/min. This result is related to the fact that an appropriate MAPP concentration could be offered as nuclei to promote the crystallization of the PP matrix.

### 3.3. Kinetic Analysis with the Avrami–Ozawa Approach

To further validate the crystallization behavior, the overall non-isothermal crystallization kinetics of the neat PP and WPCs were analyzed by the Avrami–Ozawa approach. The *F*(*T*) and *α* can be determined from the intercept and the slope, respectively, of a series of straight lines at a given relative crystallinity in the plot of ln *ϕ* against ln *t* according to Equation (8). Figure 3b presents the characteristic plot of ln *ϕ* versus ln *t* of the neat PP at various relative crystallinities. Additionally, the corresponding data for each sample are listed in Table 3. The data for each crystallization stage were fitted with a straight line, and the *R*^2^ value was greater than 0.96, indicating that this method is applicable for describing the non-isothermal crystallization kinetics of the neat PP and its composites. The *F*(*T*) of all samples steadily increased with increasing *X*, but the values of *α* were almost constant. Accordingly, a higher crystallinity degree was obtained by using a higher cooling rate at the unit crystallization time. Furthermore, the *F*(*T*) values of all WPCs were lower than those of the neat PP at a given relative crystallinity. This result indicated that a slower cooling rate can achieve a higher degree of crystallinity due to the faster crystallization of WPCs. Among all samples, the WPC_M3_ showed the lowest *F*(*T*) value at a given relative crystallinity. This is consistent with the results of the Avrami approach given above.

### 3.4. Activation Energy of Non-Isothermal Crystallization by the Kissinger Method and the Friedman Method

**Figure 4:**
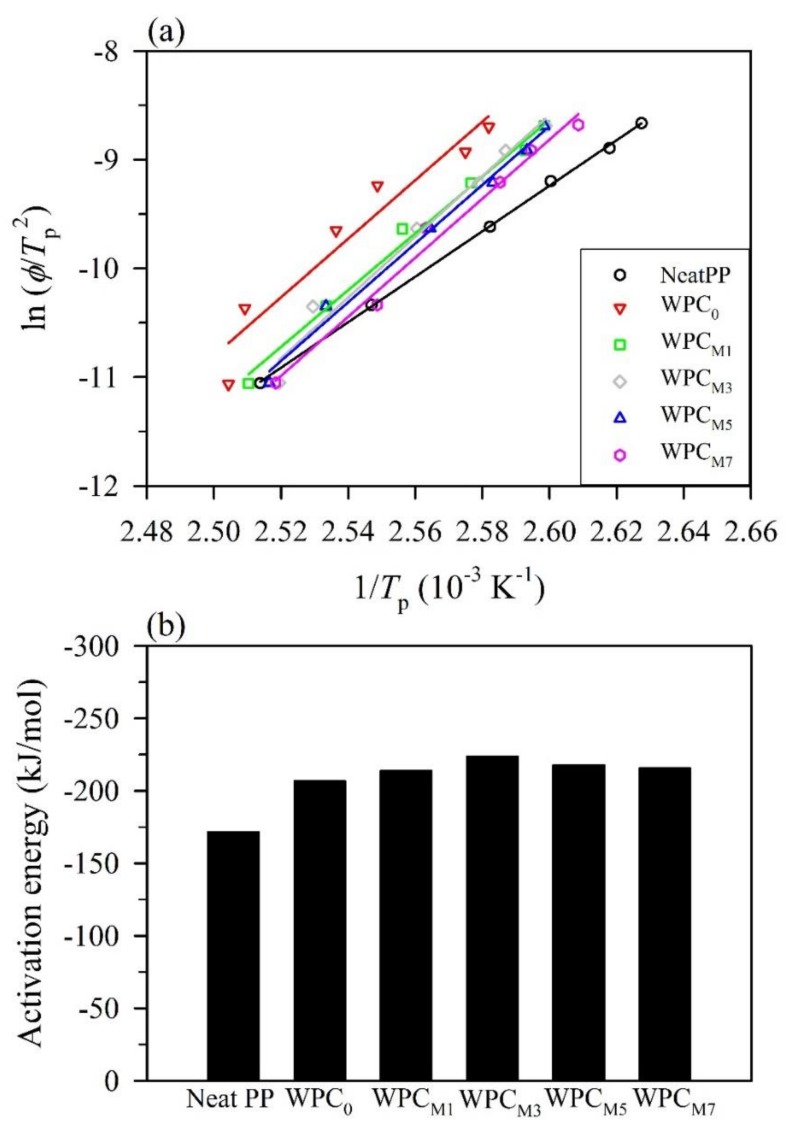
The non-isothermal crystallization kinetics of the neat PP and WPCs using the Kissinger method. (**a**) Kissinger plots of ln (*ϕ*/*T*_p_^2^) versus 1/*T*_p_^2^; (**b**) the crystallization activation energy.

One of the indispensable parameters is the crystallization activation energy in polymer systems. The crystallization of a polymer matrix in composites is influenced by the following two factors: the static factor, which is related to the free barrier energy of nucleation, and the dynamic factor, which corresponds to the activation energy for the transport of the macromolecular segments to the surface of crystal growth [49]. Among the various kinetic models, the Kissinger method has successfully been used to estimate the crystallization activation energy for polymers. The crystallization activation energy (Δ*E*) can be calculated according to the Kissinger equation (Equation (9)). Figure 4a shows the Kissinger plots of ln (*ϕ*/*T*_p_^2^) versus 1/*T*_p_^2^ for the neat PP and WPCs, enabling the determination of the Δ*E* values from the slopes of the good linear regression for all samples. Accordingly, as shown in Figure 4b, the Δ*E* value of the WPC_0_ was −207 kJ/mol, which is lower than that of the neat PP (−172 kJ/mol) due to the nucleating effect of the WFs. Clearly, the Δ*E* value was further decreased to −224 kJ/mol when 3 wt % MAPP was added to the WPC. However, the crystallization activation energy increased to −218 kJ/mol and −216 kJ/mol for the WPC_M5_ and WPC_M7_, respectively. This result indicated that optimal MAPP concentrations could accelerate the crystal growth of the polymer matrix, while excessive MAPP loading would increase the crystallization activation energy to hinder the transport of polymer chains for its crystallization, as further verified by the results described above.

**Figure 5:**
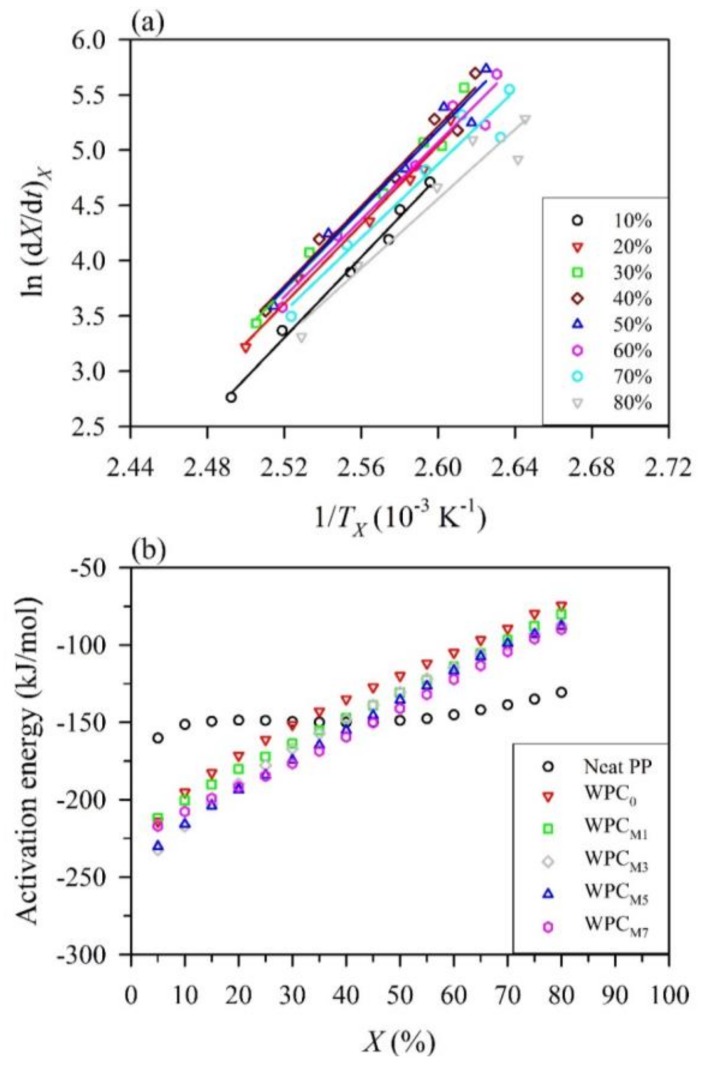
The non-isothermal crystallization kinetics of the neat PP and WPCs using the Friedman method. (**a**) Friedman plots of ln (*dX/dt*)*_X_* versus 1/*T_X_* for the neat PP; (**b**) dependence of the effective activation energy on the relative crystallinities for the neat PP and WPCs.

Furthermore, the isoconventional Friedman method was also used in this study since it is considered for non-isothermal crystallization as a function of the relative crystallinity. The ln (*dX*/*dt*)*_X_* versus 1/*T_X_* plots for the neat PP at different relative crystallinities are illustrated in Figure 5a. According to the Friedman equation (Equation (10)), the activation energies at a given relative crystallinity, *E_X_*, can be obtained from the slopes of the straight line of the Friedman plots for the neat PP and WPCs as shown in Figure 5b. The *E_X_* value of the neat PP varied in the finite range from −160 to −130 kJ/mol. In contrast, the *E_X_* values of all WPCs increased with increasing *X* from 5 to 80%. The resulting activation energies are in the ranges from −214 to −74 kJ/mol, from −212 to −80 kJ/mol, from −232 to −86 kJ/mol, from −230 to −88 kJ/mol, and from −217 to −90 kJ/mol for WPC_0_, WPC_M1_, WPC_M3_, WPC_M5_, and WPC_M7_, respectively. These findings suggest that the crystallization process easily proceeded at the initial stages of crystallization, while it became more difficult to crystallize as higher crystallinity was achieved. A similar result was observed for the short carbon fiber/poly(trimethylene terephthalate) composites [50]. Additionally, in comparison with the neat PP, a significant decrease in the *E_X_* values for all WPCs was observed when *X* was below 30%. Among them, the WPC_0_ exhibited the highest energy barrier at the given *X*, while the addition of MAPP into the WPC resulted in a decrease of the *E_X_* value in a dose-dependent manner, except at lower *X* (<20%) values. It can be noted that the *E_X_* values of the WPC_M7_ were higher than those of the WPC_M3_ and WPC_M5_ at *X* values of 5–20%. These results confirmed that MAPP could act as a heterogeneous nucleation agent to accelerate the crystallization process of the PP, but excessive MAPP loading would restrain the formation of crystals.

## 4. Conclusions

The non-isothermal crystallization kinetics of WPCs with various MAPP concentrations were determined by DSC and analyzed by the Avrami method, Avrami–Ozawa method, Kissinger method, and Friedman method. According to the Avrami method, the addition of MAPP into WPC significantly changed the nucleation mechanism of the PP matrix from homogeneous nucleation for acicular crystal growth to heterogeneous nucleation for acicular and tabular crystal growth under the cooling process. Additionally, the Avrami–Ozawa method indicated that a higher crystallinity of the PP matrix could be obtained for WPCs compared to the neat PP at a lower cooling rate, and the WPC with 3 wt % MAPP (WPC_M3_) showed the highest crystallization rate at a given relative crystallinity among all samples. Furthermore, the Kissinger method indicated that the crystallization activation energy (Δ*E*) decreased when WFs and MAPP were added to the PP matrix, and the WPC_M3_ exhibited the lowest Δ*E* value among all WPCs. On the other hand, using the iso-conversional Friedman method, the results showed that the addition of WF and MAPP easily induced the PP matrix to crystallize at the initial stages, while crystallization was difficult to achieve at a higher crystallization. Overall, these results indicated that the 3 wt % MAPP addition could optimally contribute to the acceleration of the crystal growth of the polymer matrix; however, excessive MAPP loading would hinder the crystallization of polymer chains. From these methods, it was observed that the crystallization mechanism, crystallization rate, and activation energy for the PP matrix were influenced by the cooling rate and MAPP content. The addition of different MAPP concentrations can affect the crystallization behavior of the PP by either improving or hindering crystal growth, leading to competition between heterogeneous nucleation and crystal growth in the restricted interspaces between the WFs and MAPP. Therefore, for optimizing the polymer morphology in a composite, these results offer information regarding the conditions of the manufacturing process.

## Figures and Tables

**Table 1:** Sample code and composition of the neat polypropylene (PP) and its composites.

Code	WF (wt %)	PP (wt %)	MAPP (wt %)
Neat PP	0	100	0
WPC_0_	60	40	0
WPC_M1_	60	39	1
WPC_M3_	60	37	3
WPC_M5_	60	35	5
WPC_M7_	60	33	7

**Table 2:** The parameters calculated at various cooling rates based on the Avrami model.

Code	Cooling Rate (°C/min)	*K*	*K*_J_	*n*	R^2^
Neat PP	2.5	0.19 ± 0.01	0.52 ± 0.01	1.77 ± 0.05	0.9856
	5	0.28 ± 0.02	0.78 ± 0.01	1.74 ± 0.04	0.9873
	10	0.69 ± 0.10	0.96 ± 0.01	1.67 ± 0.06	0.9823
	15	1.31 ± 0.59	1.01 ± 0.03	1.83 ± 0.02	0.9817
	20	5.17 ± 1.51	1.08 ± 0.02	1.87 ± 0.22	0.9878
	25	14.23 ± 0.52	1.11 ± 0.00	1.99 ± 0.05	0.9931
WPC_0_	2.5	0.63 ± 0.04	0.83 ± 0.02	2.04 ± 0.35	0.9889
	5	0.14 ± 0.04	0.67 ± 0.04	1.84 ± 0.00	0.9885
	10	1.08 ± 0.26	1.00 ± 0.02	1.77 ± 0.70	0.9923
	15	0.73 ± 0.03	0.98 ± 0.01	1.70 ± 0.16	0.9836
	20	1.12 ± 0.05	1.01 ± 0.02	1.73 ± 0.13	0.9854
	25	5.74 ± 1.62	1.07 ± 0.01	1.65 ± 0.32	0.9951
WPC_M1_	2.5	0.20 ± 0.04	0.52 ± 0.05	1.57 ± 0.10	0.9770
	5	0.26 ± 0.03	0.76 ± 0.02	1.62 ± 0.12	0.9776
	10	0.63 ± 0.05	0.96 ± 0.01	1.82 ± 0.29	0.9487
	15	3.08 ± 1.14	1.07 ± 0.03	1.93 ± 0.11	0.9807
	20	1.24 ± 0.39	1.01 ± 0.02	1.71 ± 0.08	0.9673
	25	1.59 ± 0.52	1.02 ± 0.01	1.93 ± 0.01	0.9925
WPC_M3_	2.5	0.79 ± 0.47	0.87 ± 0.23	2.03 ± 0.88	0.9984
	5	0.40 ± 0.01	0.83 ± 0.00	1.75 ± 0.28	0.9660
	10	1.82 ± 0.30	1.06 ± 0.02	1.61 ± 0.25	0.9655
	15	3.33 ± 0.07	1.08 ± 0.00	1.99 ± 0.09	0.9859
	20	2.27 ± 0.06	1.04 ± 0.00	1.42 ± 0.08	0.9886
	25	2.27 ± 0.09	1.03 ± 0.00	1.50 ± 0.20	0.9819
WPC_M5_	2.5	0.22 ± 0.02	0.55 ± 0.02	1.55 ± 0.03	0.9872
	5	0.16 ± 0.10	0.67 ± 0.09	1.43 ± 0.10	0.9385
	10	0.92 ± 0.21	0.99 ± 0.02	1.89 ± 0.04	0.9679
	15	0.78 ± 0.07	0.98 ± 0.01	1.40 ± 0.55	0.9711
	20	1.11 ± 0.31	1.00 ± 0.01	1.37 ± 0.27	0.9449
	25	2.77 ± 0.20	1.04 ± 0.00	1.61 ± 0.44	0.9906
WPC_M7_	2.5	0.11 ± 0.01	0.42 ± 0.01	1.56 ± 0.18	0.9697
	5	0.17 ± 0.02	0.70 ± 0.01	1.28 ± 0.03	0.9808
	10	0.63 ± 0.11	0.95 ± 0.02	1.32 ± 0.03	0.9752
	15	5.35 ± 0.44	1.12 ± 0.01	1.68 ± 0.40	0.9848
	20	1.21 ± 0.35	1.01 ± 0.02	1.77 ± 0.52	0.9476
	25	4.09 ± 1.61	1.05 ± 0.02	2.03 ± 0.22	0.9762

Values are the means ± SD (*n* = 2).

**Table 3:** The parameters calculated at various relative crystallinities (*X*) based on the Avrami–Ozawa model.

Code	*X* (%)	*F*(*T*)	*α*	R^2^
Neat PP	20	8.8 ± 0.1	1.2 ± 0.0	0.9891
	40	10.8 ± 0.1	1.1 ± 0.0	0.9952
	60	12.6 ± 0.1	1.1 ± 0.0	0.9954
	80	14.7 ± 0.1	1.1 ± 0.0	0.9941
WPC_0_	20	7.4 ± 0.2	1.0 ± 0.1	0.9611
	40	8.8 ± 0.4	1.0 ± 0.0	0.9636
	60	9.9 ± 0.4	1.1 ± 0.1	0.9687
	80	11.5 ± 0.5	1.1 ± 0.0	0.9661
WPC_M1_	20	7.6 ± 0.3	1.2 ± 0.0	0.9685
	40	9.7 ± 0.1	1.2 ± 0.1	0.9869
	60	11.1 ± 0.1	1.2 ± 0.1	0.9885
	80	12.9 ± 0.2	1.3 ± 0.1	0.9898
WPC_M3_	20	7.1 ± 0.0	1.1 ± 0.1	0.9711
	40	8.5 ± 0.1	1.1 ± 0.1	0.9765
	60	9.4 ± 0.1	1.1 ± 0.1	0.9774
	80	11.3 ± 0.2	1.2 ± 0.1	0.9786
WPC_M5_	20	7.9 ± 0.7	1.0 ± 0.1	0.9705
	40	9.3 ± 0.9	1.1 ± 0.1	0.9780
	60	10.8 ± 1.3	1.1 ± 0.1	0.9821
	80	12.4 ± 1.4	1.2 ± 0.1	0.9846
WPC_M7_	20	8.6 ± 0.2	1.2 ± 0.0	0.9818
	40	10.3 ± 0.2	1.2 ± 0.0	0.9846
	60	11.7 ± 0.2	1.2 ± 0.0	0.9853
	80	13.7 ± 0.0	1.2 ± 0.0	0.9849

Values are the means ± SD (*n* = 2).
